# Association between parental recognition and engagement in child maltreatment: an Internet-based cross-sectional study in Japan

**DOI:** 10.1265/ehpm.24-00388

**Published:** 2026-03-04

**Authors:** Miho Sodeno, Sumiyo Okawa, Mariko Hosozawa, Takahiro Tabuchi

**Affiliations:** 1 Bureau of Global Health Cooperation, Japan Institute for Health Security, 1-21-1 Toyama, Shinjuku-ku, Tokyo 162-8655, Japan; 2 Institute for Global Health Policy Research, Bureau of Global Health Cooperation, Japan Institute for Health Security, 1-21-1 Toyama, Shinjuku-ku, Tokyo 162-8655, Japan; 3 Division of Epidemiology, School of Public Health, Graduate School of Medicine, Tohoku University, 2-1 Seiryo-machi, Aoba-ku, Sendai, Miyagi 980-8575, Japan; 4 Cancer Control Center, Osaka International Cancer Institute, 3-1-69 Otemae, Chuo-ku, Osaka-shi, Osaka 541-8567, Japan

**Keywords:** Child abuse, Child maltreatment, Child neglect, Early childhood, Fathers, Parenting behavior

## Abstract

**Background:**

Child maltreatment, traditionally considered a maternal issue in Japan, increasingly involves fathers. Despite the rise in paternal participation, the recognition of and engagement in child maltreatment remains poorly investigated in Japan. We explored the association between parental recognition and engagement in maltreatment, stratified by parental sex.

**Methods:**

An Internet survey was conducted in Japan from July to August 2021, involving 8,819 parents (1,671 fathers, and 7,148 mothers) with children under 24 months of age. A self-report questionnaire assessed recognition of and engagement in maltreatment behaviors, including physical and psychological maltreatment and neglect. Poisson regression analysis estimated prevalence ratios of engagement in maltreatment behaviors by recognition status, stratified by parental sex.

**Results:**

Harsh behaviors widely recognized as maltreatment (unrecognition was less than 15% for both sexes) included beating the child (fathers: 13.5%, mothers: 6.9%), repeatedly insulting the child (fathers: 13.3%, mothers: 7.7%), and not feeding the child (fathers: 13.1%, mothers: 6.3%). Engagement in any form of maltreatment was reported by 14.2% of fathers and 9.6% of mothers in this sample. The overall prevalence of physical maltreatment was 10.5% among fathers and 5.7% among mothers. Similarly, fathers reported 6.1% overall neglect and mothers reported 1.9%. The prevalence of psychological maltreatment was 4.4% among fathers and 3.5% among mothers. For both sexes, lack of recognition of maltreatment was associated with a higher prevalence of engagement in such behaviors (fathers: adjusted prevalence ratio [aPR] 2.06, 95% confidence interval [CI] 1.43–2.99; mothers: aPR 1.88, 95% CI 1.53–2.33). This association was consistent across all subtypes, with the strongest for neglect (physical maltreatment [fathers: aPR 2.49, 95% CI 1.63–3.79; mothers: aPR 2.06, 95% CI 1.59–2.66], psychological maltreatment [fathers: aPR 2.22, 95% CI 1.30–3.78; mothers: aPR 1.43, 95% CI 1.09–1.88], and neglect [fathers: aPR 4.28, 95% CI 2.71–6.78; mothers: aPR 4.75, 95% CI 3.23–6.99]).

**Conclusions:**

Lack of recognition was associated with greater engagement in maltreatment, particularly neglect, for both parents, based on these findings. Findings underscore the need for parenting education and support targeting both parents.

**Supplementary information:**

The online version contains supplementary material available at https://doi.org/10.1265/ehpm.24-00388.

## Background

Child maltreatment (CM), as defined by the World Health Organization, includes all forms of physical, psychological, and sexual abuse; neglect; and exploitation [1]. This pervasive issue causes immediate harm to children’s physical and mental health and has long-term effects on development, including brain and cognitive function [2]. The impact of adverse childhood experiences (ACEs) continues into adulthood, increasing the risk of poor physical and mental health, behavioral issues, and impaired social functioning [3–6]. Before the coronavirus disease 2019 (COVID-19) pandemic, approximately one billion children worldwide experienced maltreatment annually [7]. In Asia, the prevalence of physical maltreatment was estimated as 13.9% in 2018 [8]. Increased parental stress and loss of support systems due to the pandemic have contributed to increased CM [9]. A recent study estimated that the global prevalence of physical maltreatment would rise to 18% by 2022, with psychological maltreatment affecting 39% of children worldwide [10]. Therefore, CM is considered a global public health concern.

In Japan, the number of CM-related consultations has tripled in the past decade [11]. Various risk factors related to parents, children, and social contexts contribute to maltreatment [12]. A unique challenge in Japan is the cultural conflation of child discipline with maltreatment, where physical punishment is traditionally viewed as a disciplinary measure. This has led to a lower awareness of what constitutes maltreatment compared with that in other countries [13]. Japanese parents who engage in CM often perceive their behavior as discipline [14–16].

Traditionally, mothers have played a primary role in childrearing in Japan, and over 60% of CM cases have been reported by mothers [17]. Consequently, past policies and studies have predominantly focused on mothers, whereas public health nurses in Japan support mothers during child health checkups [18]. Previous studies in Japan that examined the recognition of CM targeted mothers [13, 15, 19–23]. However, parenting dynamics in Japan have changed rapidly, and fathers’ parenting participation has recently increased owing to child-rearing policies [11, 24]. Although paternal participation in parenting and the rate of childcare leave have recently increased, paternal CM has also increased [25]. However, the social support for fathers is limited [26]. In Sweden, which has the longest history of paternal childcare leave, fathers are the primary child abusers [27]. Despite the rise in paternal participation in parenting, recognition and actual engagement in CM among mothers and fathers remain underexplored in Japan.

This study aimed to investigate the association between recognition and actual CM behaviors stratified by sex. We hypothesized that not recognizing CM would be associated with a higher likelihood of engaging in such behaviors. This may be explained by cognitive dissonance theory [28, 29], which suggests that individuals are less likely to alter their behaviors if they do not perceive them as problematic. Cultural norms that make it difficult to separate discipline from abuse may prevent parents from recognizing their actions as harmful, leading to continued abusive practices.

## Methods

### Study design and participants

This cross-sectional study was conducted as part of the Japan COVID-19 Society Internet Survey between July and August 2021 [30]. This study utilized a convenience sampling method from the pooled panels of Rakuten Insight, an Internet research company [31]. This panel included approximately 2.2 million voluntary registrants, who received minor incentive points upon completion of the questionnaire. The eligible participants of this study were mothers who gave birth after July 2019 or were expected to give birth by December 2021, and fathers whose partners were pregnant or postpartum within 24 months. First, an Internet research company conducted a screening survey targeting 440,323 potentially eligible participants and identified 14,086 mothers (2,425 pregnant and 11,661 postpartum mothers) and 3,346 fathers who met the eligibility criteria.

Next, the internet research company emailed the questionnaire to all eligible panelists and received responses from 10,000 individuals, comprising 8,047 mothers (response rate: 57.1%) and 1,953 fathers (response rate: 56.8%) between July 28 and August 30, 2021. Data collection stopped after 10,000 responses were obtained (mothers: 57.1%; fathers: 56.8%).

**Fig. 1:**
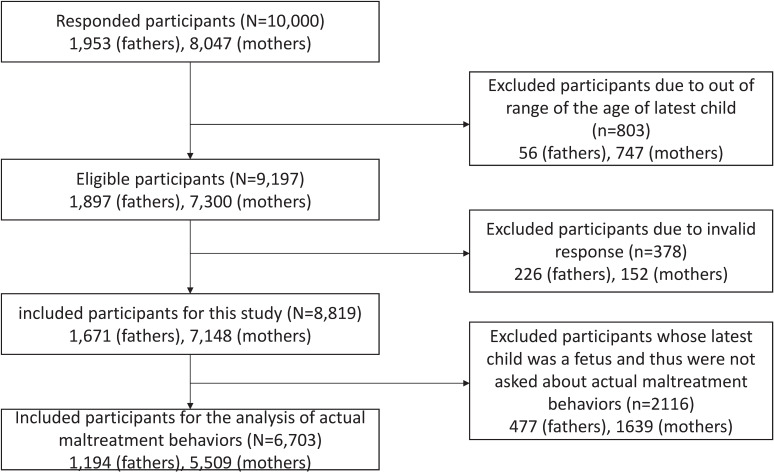
Flow chart of the study participants

We excluded 803 participants (747 mothers and 56 fathers) whose youngest children were outside the 24-month target age and 378 respondents (152 mothers and 226 fathers) with invalid responses using previously published criteria [32]. Finally, 8,819 participants (7,148 mothers and 1,671 fathers) were included (Fig. 1). Pregnant and postpartum parents were included in the questions regarding recognition of maltreatment. For maltreatment behaviors (i.e., the study outcome) and the neonatal intensive care unit (NICU) admission of the youngest child, only respondents with children under 24 months of age were deemed eligible to respond to the questions (6,703 participants: 5,509 mothers and 1,194 fathers).

### Measurement

#### Maltreatment behaviors (primary outcome)

Maltreatment behaviors were assessed using a self-reported questionnaire. We included eight items assessing CM by parents, including physical maltreatment (“hitting the child’s body” and “beating the child”), psychological maltreatment (“having a big fight in front of the child” and “insulting the child repeatedly”), neglect (“shutting the child outside,” “leaving the child alone in the house at night,” and “not feeding the child”), and other forms of ill-treatment (“smoking in front of the child”). These items are based on the CM Scale developed in Japan [33–35], and “smoking in front of the child” was added based on its importance considering the public health aspect, evidence from prior studies characterizing frequent secondhand smoke as child maltreatment [36–38], and policy recommendations by the Japan Pediatric Society [39, 40]. We excluded “yelling at the child” and “ignoring the child” based on cultural considerations: in the Japanese context, these behaviors may be interpreted as discipline rather than CM depending on their frequency, severity, and context [35, 41, 42]. Parents reported each behavior frequency on a four-point Likert scale (1 = often; 2 = sometimes; 3 = rarely; 4 = not at all). Following an expert review of a previous study, we dichotomized the responses by considering the severity and frequency of maltreatment behaviors [33] (Table 1). Unlike the previous study, which used a uniform cutoff (i.e., categorizing only “often” as “yes”), we did not apply a single threshold across all behaviors. Instead, the dichotomization into “yes” and “no” was determined through careful discussion among the research team, considering both the nature of the behavior and its distribution in the sample. The cut-off was set accordingly to ensure analytical stability and interpretability. For instance, for severe forms of CM that were rarely endorsed by participants—where the combined proportion of “often,” “sometimes,” and “rarely” responses was less than 5%—these three response categories were grouped together and categorized as “yes.” Overall physical maltreatment was considered to exist when at least one item on physical maltreatment behaviors was categorized as “Yes” in Table 1. The same coding scheme applied to psychological maltreatment, neglect, and overall child maltreatment.

**Table 1:** Dichotomized responses of CM behaviors with a four-point Likert scale

**Category**	**Maltreatment behaviors** **(The participants were asked: currently, do you ** **perform any of the following actions at home?)**	**Definition for maltreatment**			

		**1 =** **often**	**2 =** **sometimes**	**3 =** **rarely**	**4 =** **not at all**
Physical maltreatment	Hit the child’s body (buttocks, hand, head, or face)	Yes	Yes	No	No
	Beat the child	Yes	Yes	Yes	No
Psychological maltreatment	Have a big fight in front of the child	Yes	Yes	No	No
	Insult the child repeatedly	Yes	Yes	No	No
Neglect	Shut the child outside	Yes	Yes	Yes	No
	Leave the child alone in the house at night	Yes	Yes	Yes	No
	Do not feed the child	Yes	Yes	Yes	No
Other forms of ill-treatment	Smoking in front of the child	Yes	No	No	No

#### Recognition of maltreatment (primary exposure)

Parental recognition of maltreatment was assessed by asking “Do you perceive the following behaviors toward your children as maltreatment?”. The behaviors assessed included the following: hitting the child’s buttocks, hitting the child’s head, hitting the child’s face, beating the child, having a big fight in front of the child, repeatedly insulting the child, shutting out the child, leaving the child alone in the house at night, not feeding the child, and smoking in front of the child. These items were developed by our research team based on self-reported maltreatment behaviors. We examined the recognition of “hitting the child’s body” through three specific questions: hitting the child’s buttocks, hitting the child’s head, and hitting the child’s face. This approach allowed us to assess the recognition of hitting behavior concretely. We excluded “yelling at the child” and “ignoring the child” because, without context, some respondents might consider these behaviors as discipline.

These 10 items were classified as physical maltreatment, psychological maltreatment, neglect, and other maltreatment following the same rules as stipulated for maltreatment behaviors: physical maltreatment (“hitting the child’s buttocks,” “hitting the child’s head,” “hitting the child’s face,” and “beating the child”), psychological maltreatment (“having a big fight in front of the child” and “insulting the child repeatedly”), neglect (“shutting the child outside,” “leaving the child alone in the house at night,” and “not feeding the child”), and other forms of ill-treatment (“smoking in front of the child”). Physical maltreatment was considered present if any of the four physical-maltreatment items were classified as “yes.” Similarly, psychological maltreatment and neglect were considered to be present if any of the items classified under their respective categories were present. These classifications were referred to as overall physical maltreatment, psychological maltreatment, and overall neglect.

#### Covariates

We included parent- and child-related variables as potential covariates that could affect parents’ maltreatment behaviors [12].

Parental age at survey was dichotomized at the median (35 years or older and below 35 years) to ensure a balanced comparison between the groups. Parental educational attainment was categorized into two groups (college or higher, and other).

Parental psychological distress was measured using the Japanese version of the Kessler 6 scale (range: 0–24; higher scores indicated higher levels of psychological distress) [43, 44]. The participants were dichotomized into two groups (scores <10, ≥10), based on the cutoff used in the national representative survey, “Comprehensive Survey of Living Conditions,” conducted by the Ministry of Health, Labor and Welfare (MHLW) [45].

ACEs were assessed by using The Japanese ACE scale [46]. The scale consists of the following 10 items: parents died, parents were divorced or separated, a parent had a mental illness, father was violent with the mother, was severely beaten and injured in childhood by a parent, did not receive necessary care such as feeding or dressing, parents said hurtful things or were insulted, faced economic hardship, bullied at school, and sexually touched by an adult. Participants were classified as having no ACEs or at least one ACE.

Experience of domestic violence (DV) was assessed by asking whether the respondents had experienced any of the following from a partner: huge fights, physical violence, insults, injury, financial abuse, sexual abuse, or concern for unwanted pregnancy. Participants were classified based on whether they had disclosed one or more DV experiences (no DV or history of DV).

Child-related factors included age at the time of the survey (fetus, infant: 0–12 months; toddler: 1–2 years), siblings of the youngest child (yes or no), and NICU admission at birth (yes or no). The question on NICU admission was asked only of those who had or whose partners had delivered their youngest child (N = 6,703).

### Ethical approval and consent from participants

This study was approved by the Institutional Review Board of Osaka International Cancer Institute, Japan (approval number: 20084). The surveys were conducted anonymously. Informed consent was obtained electronically, and all participants were informed of their right to withdraw from the study.

### Statistical analyses

First, we calculated and compared the descriptive statistics for participant characteristics according to parental sex using a chi-square test or t-test. Second, we calculated the proportion of maltreatment behaviors and their recognition stratified by parental sex. Third, we calculated prevalence ratios (PRs) using multivariate Poisson regression with robust standard errors to examine the association between maltreatment recognition and behavior. The analysis was stratified by sex due to the sample size difference, and the model was adjusted for parental age, level of education, psychological distress (K6 ≥10), ACEs, DV experience, age of the youngest child, presence of siblings, and NICU admission. Marital status was excluded as a covariate because all fathers in the dataset were married, resulting in no variation. The data were analyzed using Stata version 15.1 (StataCorp, College Station, TX, USA). Statistical significance was set at a two-sided p-value of less than 0.05.

## Results

The sociodemographic characteristics of the participants are presented in Table 2. The mean age of the participants was 35.4 years (standard deviation [SD] 5.2) for the fathers and 32.0 (SD 4.4) for the mothers. Postpartum mothers accounted for 77.1% of all mothers, and 71.4% of fathers had partners in the postpartum period at the time of the survey. The parameters that differed significantly by parental sex were age, marital status, educational level, ACEs, DV experiences, age of the youngest child, and siblings of the youngest child, with mothers reporting more ACEs and DV experiences than did fathers.

**Table 2:** Basic characteristics of participating parents

**Variables**	**Total** **(n = 8819)** **(%)**	**Fathers** **(n = 1671)** **(%)**	**Mothers** **(n = 7148)** **(%)**	p-value for group difference*
Mean age, years (SD)	32.7 (4.8)	35.4 (5.2)	32.0 (4.4)	<0.001
Age group				<0.001
Under 35 years	5857 (66.4)	771 (46.1)	5086 (71.2)	
35 years and older	2962 (33.6)	900 (53.9)	2062 (28.9)	
Marital status				<0.001
Married	8713 (98.8)	1671 (100.0)	7042 (98.5)	
Single/divorced/widow	106 (1.2)	0 (0.0)	106 (1.5)	
Level of education				<0.001
Under high school graduation	1511 (17.1)	227 (13.6)	1284 (18.0)	
Over college graduation	7308 (82.9)	1444 (86.4)	5864 (82.0)	
Psychological distress (K6 score^a^)				0.804
K6 score <10	5881 (66.7)	1110 (66.4)	4771 (66.8)	
K6 score ≥10	2938 (33.3)	561 (33.6)	2377 (33.3)	
Adverse child experiences (ACEs)				<0.001
No ACEs	4792 (54.3)	1054 (63.1)	3738 (52.3)	
With ACEs	4027 (45.7)	617 (36.9)	3410 (47.7)	
Domestic violence (DV) experience				<0.001
No history of DV	5981 (67.8)	1214 (72.7)	4767 (66.7)	
With a history of DV	2838 (32.2)	457 (27.4)	2381 (33.3)	
Age of the youngest child				
Fetus	2116 (24.0)	477 (28.6)	1639 (22.9)	<0.001
Infant (0–11 months)	4338 (49.2)	756 (45.2)	3582 (50.1)	
Toddler (12–24 months)	2365 (26.8)	438 (26.2)	1927 (27.0)	
Siblings for the youngest child				<0.001
No siblings (first born)	4648 (52.7)	791 (47.3)	3857 (54.0)	
With one or more siblings	4171 (47.3)	880 (52.7)	3291 (46.0)	
Neonatal intensive care unit admission**				0.290
No	6148 (91.7)	1086 (91.0)	5062 (91.9)	
Yes	555 (8.3)	108 (9.1)	447 (8.1)	

^(a)^K6 score represents the Kessler 6 scale score. *Mean age was compared using the t-test, while other variables were analyzed using the chi-squared test. **NICU admission only asked for their youngest children.

**Fig. 2:**
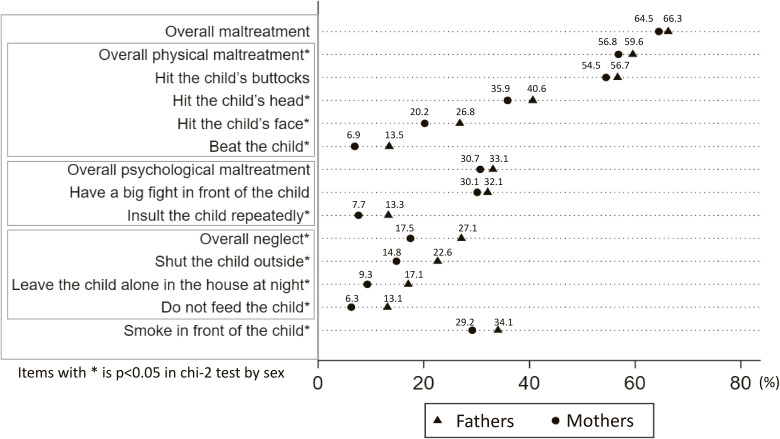
Percentage of respondents not recognizing commonly defined maltreatment behaviors, stratified by sex

Figure 2 shows the percentage of respondents who did not recognize commonly defined maltreatment behaviors, stratified by sex. Recognition of physical maltreatment was reported by 59.6% of fathers and 56.8% of mothers. Overall, neglect unrecognition was observed in 27.1% of the fathers and 17.5% of the mothers. Harsh behaviors widely recognized as maltreatment (unrecognition less than 15%) included beating the child (fathers: 13.5%, mothers: 6.9%), repeatedly insulting the child (fathers: 13.3%, mothers: 7.7%), and not feeding the child (fathers: 13.1%, mothers: 6.3%).

**Fig. 3:**
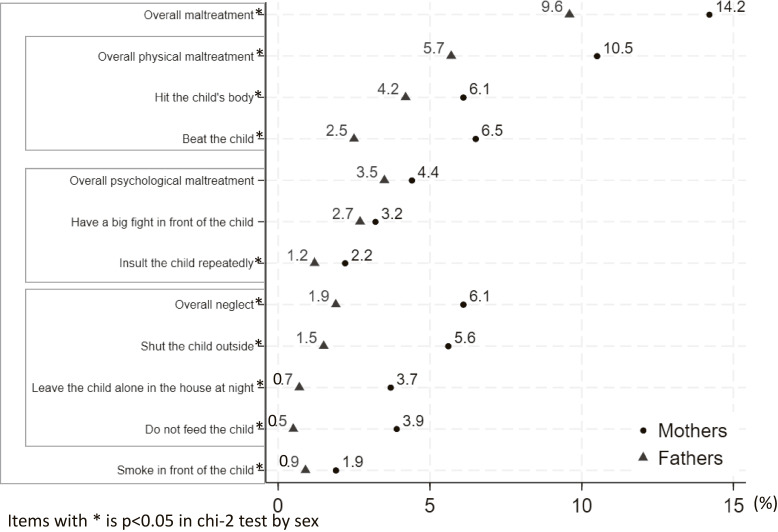
Prevalence of engagement in CM behaviors, stratified by sex

Figure 3 shows the prevalence of CM stratified according to sex. Full distributions of response categories are presented in Table S1. Overall, the CM was lower than 15% (Overall CM for fathers: 14.2%, mothers: 9.6%). Physical maltreatment prevalence was 10.5% among fathers and 5.7% among mothers. Similarly, fathers reported 6.1% overall neglect, and mothers reported 1.9%. The prevalence of psychological maltreatment was 4.4% among fathers and 3.5% among mothers.

Table 3 presents the adjusted PRs (aPRs) of the association between maltreatment recognition and behavior stratified by parental sex. After adjusting for potential covariates, fathers (aPR 2.06, 95% confidence intervals [CI] 1.43–2.99) and mothers (aPR 1.88, 95% CI 1.53–2.32) with unrecognition of any type of maltreatment showed significantly higher aPRs than those in parents who showed recognition. The lack of recognition of each subtype of maltreatment was associated with higher aPRs, with neglect showing the highest association with fathers and mothers (aPR for neglect [fathers]: aPR 4.28, 95% CI 2.70–6.78, [mothers]: aPR 4.75, 95% CI 3.22–6.99). The other subtypes of maltreatment showed similar associations (physical maltreatment [fathers]: aPR 2.49, 95% CI 1.63–3.79, [mothers]: aPR 2.06, 95% CI 1.59–2.66; psychological maltreatment [fathers]: aPR 2.22, 95% CI 1.30–3.78, [mothers]: aPR 1.43, 95% CI 1.09–1.88; neglect [fathers]: aPR 5.52, 95% CI 2.87–10.63, [mothers]: aPR 3.25, 95% CI 1.99–5.29). The results of the associations between the other adjusted variables and maltreatment are provided in Tables S2–5.

**Table 3:** Association between parental maltreatment behaviors and recognition status, stratified by parental sex

**Parental recognition status ** **for maltreatment**	**Fathers (n = 1,194)**	**Mothers (n = 5,509)**
	**aPR (95%CI)**	**aPR (95%CI)**
Any maltreatment behaviors		
Recognized	Ref	Ref
Not recognized	2.06 (1.43–2.99)	1.88 (1.53–2.32)

Types of maltreatment behavior		

Physical maltreatment behaviors		
Recognized	Ref	Ref
Not recognized	2.49 (1.63–3.79)	2.06 (1.59–2.66)

Psychological maltreatment behaviors		
Recognized	Ref	Ref
Not recognized	2.22 (1.30–3.78)	1.43 (1.09–1.88)

Neglect behaviors		
Recognized	Ref	Ref
Not recognized	4.28 (2.71–6.78)	4.75 (3.23–6.99)

aPR: Adjusted Prevalence Ratio. 95% CI: 95% confidence interval

Note: The models were adjusted for parental age, education level, psychological distress, adverse childhood experience, domestic violence experience, age of the youngest child, siblings of the youngest child, and NICU admission of the youngest child.

## Discussion

The primary aim of our study was to examine the relationship between parental recognition of CM and actual behavior. While the convenience sampling design limits the generalizability of prevalence estimates, this study serves as a critical step in generating hypotheses regarding the association between recognition and behavior. We observed that lack of recognition was significantly associated with a higher likelihood of engaging in all types of maltreatment behaviors, particularly neglect, for both fathers and mothers. Our findings suggest that a lack of recognition of maltreatment behaviors may increase the risk of engaging in such behaviors among both fathers and mothers participating in this study.

The findings of this study have significant implications in clinical practice and policies. One of the most important implications of this study is the need for targeted prevention programs to increase parental awareness of CM, particularly neglect. The observed association between lack of recognition and neglectful behaviors aligns with those in previous studies, highlighting the critical role of parental awareness in preventing child neglect [6]. Educational materials and interventions should be designed to help parents recognize harmful behaviors early and should be provided through local government child support centers, including health centers [15]. In Japan, local governments offer free child health checkups at 3–4, 6–7, and 9–10 months of age, with attendance rates exceeding 90% [47]. This high participation rate suggests that these check-ups provide an excellent opportunity to monitor children’s development, assess their interactions with parents, and improve their recognition of CM. According to the 2020 Japanese Welfare Policy Report from the MHLW, 1–3 years is the most frequently reported age for CM [48]. This age range is often called the “terrible twos” because toddlers show greater independence. In Japan, free child health checkups at 1.6 years of age may provide another excellent opportunity to screen for CM.

Additionally, our sex-stratified analysis revealed that lower recognition of maltreatment was associated with a higher likelihood of engaging in such behaviors for both fathers and mothers. However, the current lack of official/professional support (e.g., parenting programs, counseling services) specifically designed for fathers, compared with mothers, leaves them more vulnerable to engaging in harmful behaviors. There are psychological and physical differences between fathers and mothers that may influence their perceptions and behaviors related to parenting. For example, fathers may experience higher stress levels because of societal expectations of being the primary breadwinner, which can increase the likelihood of frustration and aggression toward their children [49, 50]. Biologically, hormonal differences between fathers and mothers may contribute to this, as fathers may exhibit a lower threshold for aggression under stress [51].

In Japan, previous studies have focused on mothers and fathers who often have fewer structured opportunities to access parenting resources, mental health services, and peer support networks [13, 15, 18–23, 52, 53]. Many local governments and maternity hospitals in Japan offer prenatal education classes to prepare parents for birth and parenting [54]. However, these classes mainly focused on birth preparation and infant care and should be expanded to include information on CM [54]. During the postpartum period, mothers receive mental health checkups and support through infant health checks and home visits. However, fathers have few such opportunities despite growing concerns about paternal depression in Japan [25]. Moreover, 98% of municipalities recognized the need to support fathers in parenting, as shown in a study in 2023; however, only 6.9% promoted fathers’ participation in child health checkups, and 5.2% provided targeted leaflets for fathers. This study revealed that over 80% of the municipalities offered no specific support to fathers [55]. Given that unrecognized maltreatment behaviors may lead both fathers and mothers to engage in such behaviors, policymakers should revise the current frameworks to ensure that both parents, who share the rights and responsibilities of childrearing, have equal access to supportive systems that foster awareness, recognition, and prevention of CM.

This study had certain limitations. First, there is a possibility of reporting bias due to the nature of self-administered surveys. Parents may underreport their maltreatment behaviors. Second, because we used data from a web-based survey, the prevalence ratios observed in this study cannot be generalized to the general population of parents in Japan due to the inherent selection bias in the convenience sampling method. Previous studies have pointed out that internet-based convenience samples may have inherent selection biases, as access to and participation in online surveys are often influenced by participants’ demographic and socioeconomic characteristics. For example, the educational level of the study participants was higher than that observed in nationally representative samples [45], which could have biased the results. Third, our study was not able to compare the association between recognition and behavior between mothers and fathers due to imbalanced sample size and the potential differences in respondent characteristics between fathers and mothers in this study. Additionally, the data for fathers and mothers were not collected from the same families, and the sex-stratified results cannot be interpreted as a direct comparison between mothers and fathers in the same conditions. Future research using a more balanced and sufficiently large sample to explore potential sex-based effect modification in parental behavior recognition would offer additional evidence for effective intervention by parental sex. Lastly, given the nature of cross-sectional studies, the association between exposure and outcomes should not be interpreted as a causal inference. Further longitudinal research with a representative sample is needed to identify the nature of this relationship.

## Conclusions

Our study highlights the significant risk posed by unrecognized maltreatment among the participating parents in Japan, with unrecognized neglect showing the strongest association with actual maltreatment behaviors. These findings underscore the need for approaches targeting both parents to improve parental recognition of maltreatment. Integrating education programs on maltreatment recognition during prenatal educational classes and regular child health checkups and expanding support programs that include both parents can be vital in reducing the incidence of CM. In Japan, however, support for fathers remains limited compared to support for mothers. Efforts should be made to strengthen support systems such that parents have equal access to parenting resources, educational opportunities, and mental health services to ensure that they are equally equipped to prevent child maltreatment.

## Abbreviations

ACE: adverse childhood experience
CI: confidence interval
CM: child maltreatment
COVID-19: coronavirus disease 2019
DV: domestic violence
MHLW: Ministry of Health, Labour and Welfare
aPR: adjusted prevalence ratio

## Supplementary information

**Additional file 1:** **Table S1.** Distribution of responses for each CM behavior across four response categories, by parental sex (detailed results for Figure 3).

**Additional file 2:** **Table S2.** Association between parental maltreatment behaviors and recognition status for overall maltreatment by parental sex: any type of maltreatment (expanded results from Table 3).

**Additional file 3:** **Table S3.** Association between parental maltreatment behaviors and recognition status for subtypes by parental sex: physical maltreatment (expanded results from Table 3).

**Additional file 4:** **Table S4.** Association between parental maltreatment behaviors and recognition status for subtypes by parental sex: psychological maltreatment (expanded results from Table 3).

**Additional file 5:** **Table S5.** Association between parental maltreatment behaviors and recognition status for subtypes by parental sex: Neglect (expanded results from Table 3).

## Declarations

### Availability of data and material

The data used in this study from the Japan COVID-19 Society Internet Survey (JACSIS) can be accessed by contacting the principal investigator as indicated via the JACSIS study website: https://jacsis-study.jp/dug/index.html.
