# Supplementary material for: Association between parental recognition and engagement in child maltreatment: an Internet-based cross-sectional study in Japan
Source: Environ Health Prev Med. 2026 Mar 4;31:15. doi: 10.1265/ehpm.24-00388 (PMC12981977; doi:10.1265/ehpm.24-00388)
Supplement: Supplementary file 1 — Additional file 1: Table S1. Distribution of responses for each CM behavior across four response categories, by parental sex (detailed results for Figure 3). [file ehpm-31-015-s001.pptx]

## Slide 1
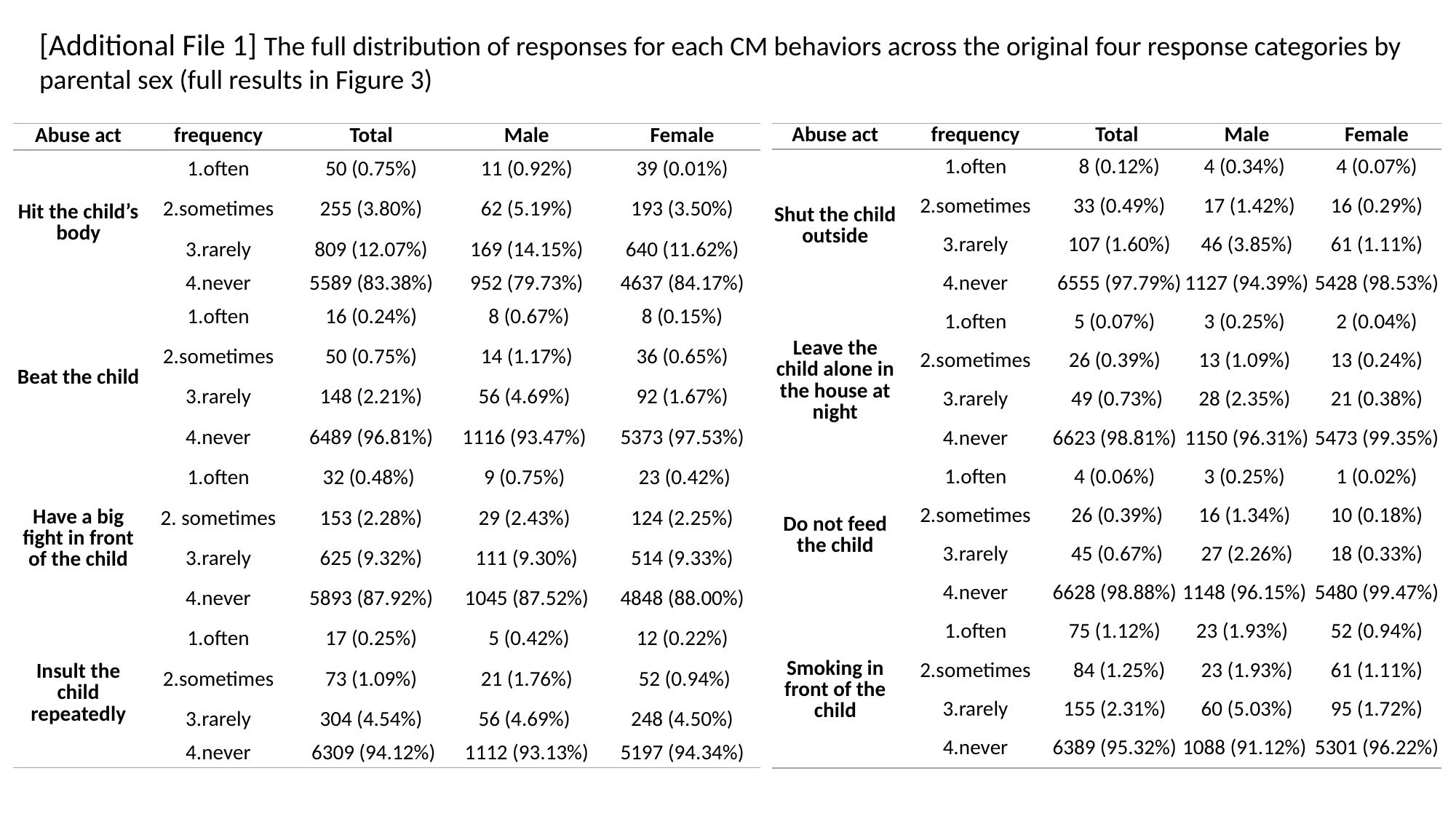

[Additional File 1] The full distribution of responses for each CM behaviors across the original four response categories by parental sex (full results in Figure 3)
| Abuse act | frequency | Total | Male | Female |
| --- | --- | --- | --- | --- |
| Hit the child’s body | 1.often | 50 (0.75%) | 11 (0.92%) | 39 (0.01%) |
| | 2.sometimes | 255 (3.80%) | 62 (5.19%) | 193 (3.50%) |
| | 3.rarely | 809 (12.07%) | 169 (14.15%) | 640 (11.62%) |
| | 4.never | 5589 (83.38%) | 952 (79.73%) | 4637 (84.17%) |
| Beat the child | 1.often | 16 (0.24%) | 8 (0.67%) | 8 (0.15%) |
| | 2.sometimes | 50 (0.75%) | 14 (1.17%) | 36 (0.65%) |
| | 3.rarely | 148 (2.21%) | 56 (4.69%) | 92 (1.67%) |
| | 4.never | 6489 (96.81%) | 1116 (93.47%) | 5373 (97.53%) |
| Have a big fight in front of the child | 1.often | 32 (0.48%) | 9 (0.75%) | 23 (0.42%) |
| | 2. sometimes | 153 (2.28%) | 29 (2.43%) | 124 (2.25%) |
| | 3.rarely | 625 (9.32%) | 111 (9.30%) | 514 (9.33%) |
| | 4.never | 5893 (87.92%) | 1045 (87.52%) | 4848 (88.00%) |
| Insult the child repeatedly | 1.often | 17 (0.25%) | 5 (0.42%) | 12 (0.22%) |
| | 2.sometimes | 73 (1.09%) | 21 (1.76%) | 52 (0.94%) |
| | 3.rarely | 304 (4.54%) | 56 (4.69%) | 248 (4.50%) |
| | 4.never | 6309 (94.12%) | 1112 (93.13%) | 5197 (94.34%) |
| Abuse act | frequency | Total | Male | Female |
| --- | --- | --- | --- | --- |
| Shut the child outside | 1.often | 8 (0.12%) | 4 (0.34%) | 4 (0.07%) |
| | 2.sometimes | 33 (0.49%) | 17 (1.42%) | 16 (0.29%) |
| | 3.rarely | 107 (1.60%) | 46 (3.85%) | 61 (1.11%) |
| | 4.never | 6555 (97.79%) | 1127 (94.39%) | 5428 (98.53%) |
| Leave the child alone in the house at night | 1.often | 5 (0.07%) | 3 (0.25%) | 2 (0.04%) |
| | 2.sometimes | 26 (0.39%) | 13 (1.09%) | 13 (0.24%) |
| | 3.rarely | 49 (0.73%) | 28 (2.35%) | 21 (0.38%) |
| | 4.never | 6623 (98.81%) | 1150 (96.31%) | 5473 (99.35%) |
| Do not feed the child | 1.often | 4 (0.06%) | 3 (0.25%) | 1 (0.02%) |
| | 2.sometimes | 26 (0.39%) | 16 (1.34%) | 10 (0.18%) |
| | 3.rarely | 45 (0.67%) | 27 (2.26%) | 18 (0.33%) |
| | 4.never | 6628 (98.88%) | 1148 (96.15%) | 5480 (99.47%) |
| Smoking in front of the child | 1.often | 75 (1.12%) | 23 (1.93%) | 52 (0.94%) |
| | 2.sometimes | 84 (1.25%) | 23 (1.93%) | 61 (1.11%) |
| | 3.rarely | 155 (2.31%) | 60 (5.03%) | 95 (1.72%) |
| | 4.never | 6389 (95.32%) | 1088 (91.12%) | 5301 (96.22%) |
