# Supplementary material for: Association between parental recognition and engagement in child maltreatment: an Internet-based cross-sectional study in Japan
Source: Environ Health Prev Med. 2026 Mar 4;31:15. doi: 10.1265/ehpm.24-00388 (PMC12981977; doi:10.1265/ehpm.24-00388)
Supplement: Supplementary file 2 — Additional file 2: Table S2. Association between parental maltreatment behaviors and recognition status for overall maltreatment by parental sex: any type of maltreatment (expanded results from Table 3). [file ehpm-31-015-s002.pptx]

## Slide 1
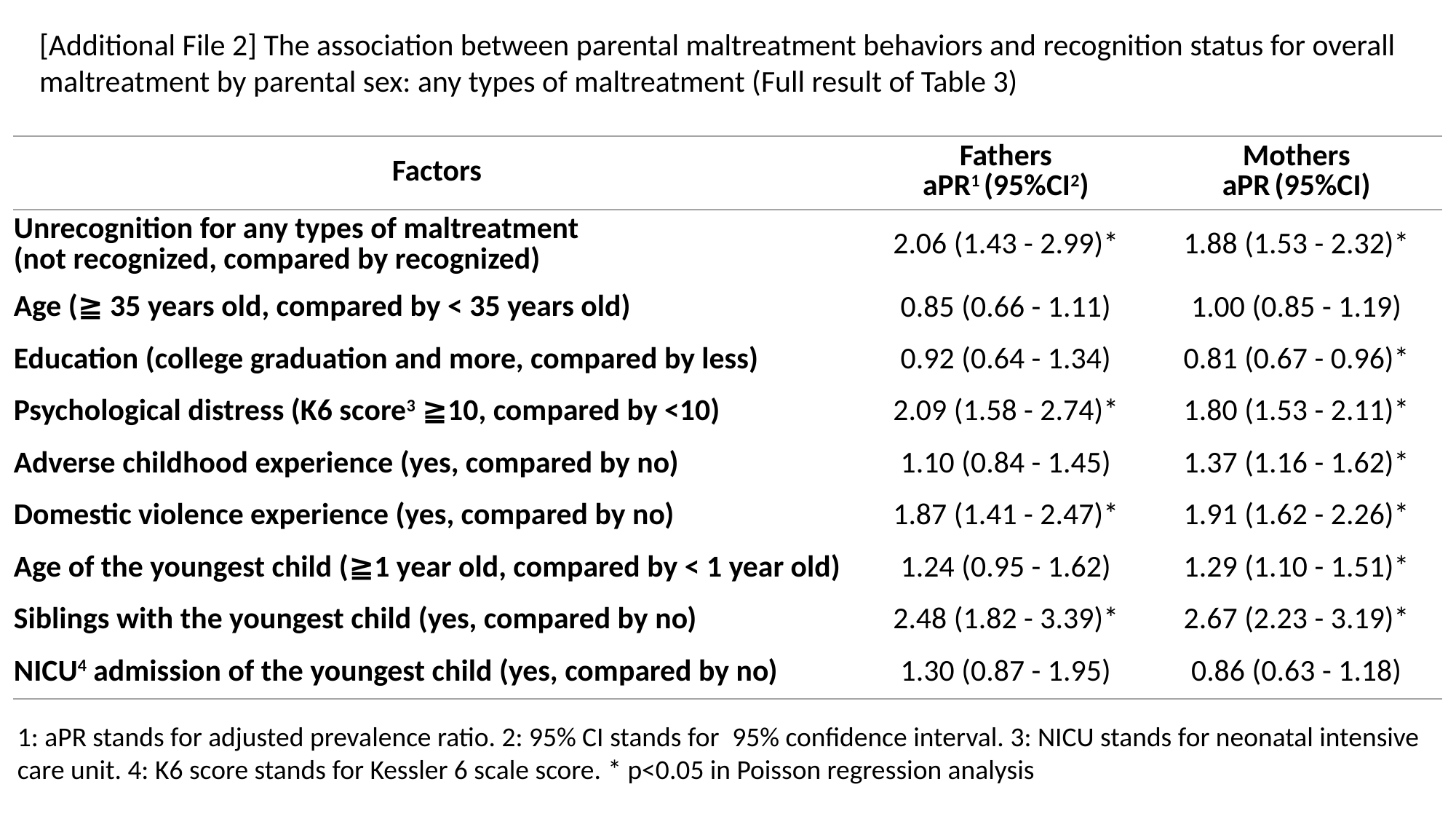

[Additional File 2] The association between parental maltreatment behaviors and recognition status for overall maltreatment by parental sex: any types of maltreatment (Full result of Table 3)
| Factors | Fathers aPR1 (95%CI2) | Mothers aPR (95%CI) |
| --- | --- | --- |
| Unrecognition for any types of maltreatment (not recognized, compared by recognized) | 2.06 (1.43 - 2.99)\* | 1.88 (1.53 - 2.32)\* |
| Age (≧ 35 years old, compared by < 35 years old) | 0.85 (0.66 - 1.11) | 1.00 (0.85 - 1.19) |
| Education (college graduation and more, compared by less) | 0.92 (0.64 - 1.34) | 0.81 (0.67 - 0.96)\* |
| Psychological distress (K6 score3 ≧10, compared by <10) | 2.09 (1.58 - 2.74)\* | 1.80 (1.53 - 2.11)\* |
| Adverse childhood experience (yes, compared by no) | 1.10 (0.84 - 1.45) | 1.37 (1.16 - 1.62)\* |
| Domestic violence experience (yes, compared by no) | 1.87 (1.41 - 2.47)\* | 1.91 (1.62 - 2.26)\* |
| Age of the youngest child (≧1 year old, compared by < 1 year old) | 1.24 (0.95 - 1.62) | 1.29 (1.10 - 1.51)\* |
| Siblings with the youngest child (yes, compared by no) | 2.48 (1.82 - 3.39)\* | 2.67 (2.23 - 3.19)\* |
| NICU4 admission of the youngest child (yes, compared by no) | 1.30 (0.87 - 1.95) | 0.86 (0.63 - 1.18) |
1: aPR stands for adjusted prevalence ratio. 2: 95% CI stands for 95% confidence interval. 3: NICU stands for neonatal intensive care unit. 4: K6 score stands for Kessler 6 scale score. * p<0.05 in Poisson regression analysis
