# Supplementary material for: Association between parental recognition and engagement in child maltreatment: an Internet-based cross-sectional study in Japan
Source: Environ Health Prev Med. 2026 Mar 4;31:15. doi: 10.1265/ehpm.24-00388 (PMC12981977; doi:10.1265/ehpm.24-00388)
Supplement: Supplementary file 3 — Additional file 3: Table S3. Association between parental maltreatment behaviors and recognition status for subtypes by parental sex: physical maltreatment (expanded results from Table 3). [file ehpm-31-015-s003.pptx]

## Slide 1
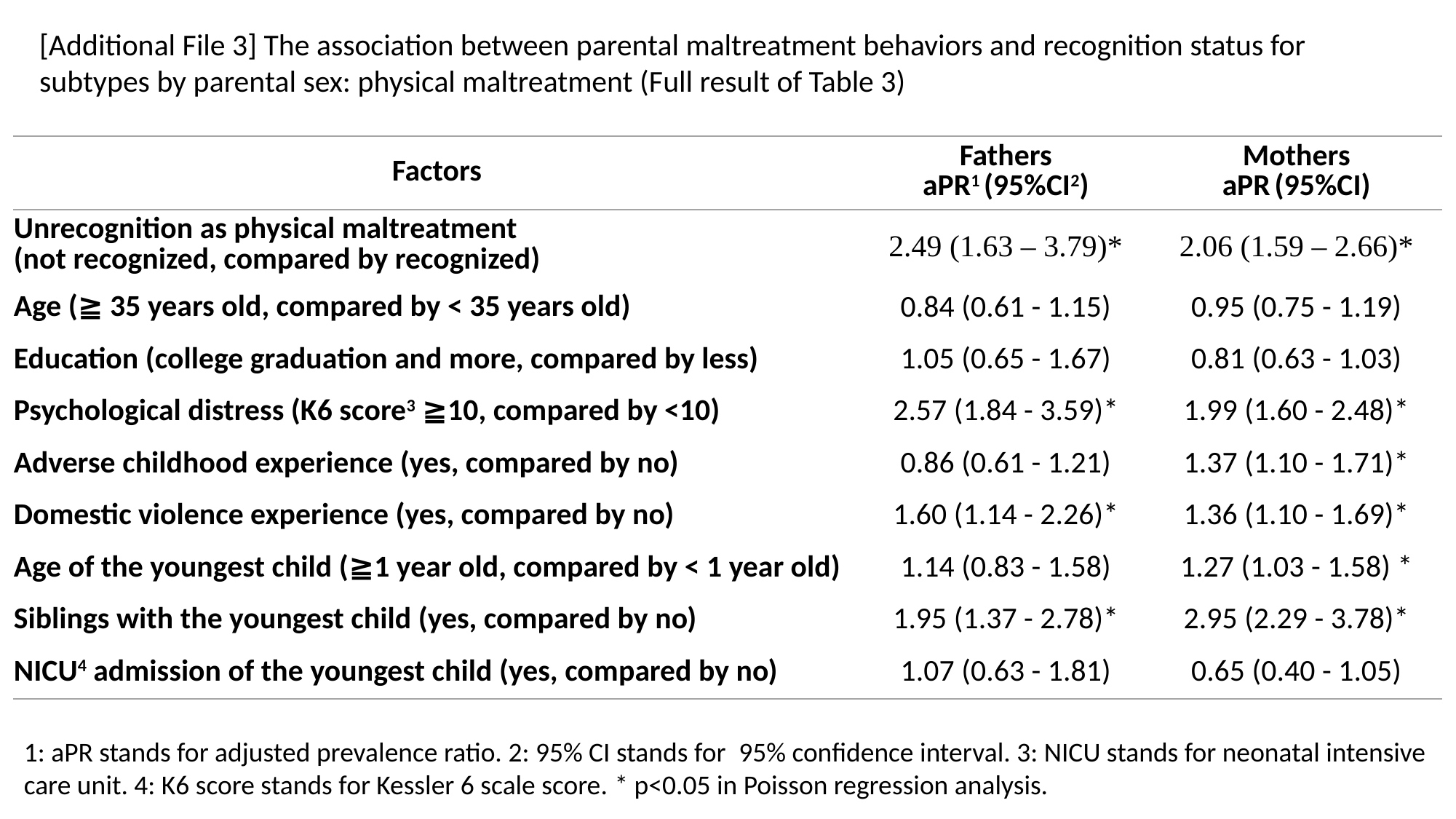

[Additional File 3] The association between parental maltreatment behaviors and recognition status for subtypes by parental sex: physical maltreatment (Full result of Table 3)
| Factors | Fathers aPR1 (95%CI2) | Mothers aPR (95%CI) |
| --- | --- | --- |
| Unrecognition as physical maltreatment (not recognized, compared by recognized) | 2.49 (1.63 – 3.79)\* | 2.06 (1.59 – 2.66)\* |
| Age (≧ 35 years old, compared by < 35 years old) | 0.84 (0.61 - 1.15) | 0.95 (0.75 - 1.19) |
| Education (college graduation and more, compared by less) | 1.05 (0.65 - 1.67) | 0.81 (0.63 - 1.03) |
| Psychological distress (K6 score3 ≧10, compared by <10) | 2.57 (1.84 - 3.59)\* | 1.99 (1.60 - 2.48)\* |
| Adverse childhood experience (yes, compared by no) | 0.86 (0.61 - 1.21) | 1.37 (1.10 - 1.71)\* |
| Domestic violence experience (yes, compared by no) | 1.60 (1.14 - 2.26)\* | 1.36 (1.10 - 1.69)\* |
| Age of the youngest child (≧1 year old, compared by < 1 year old) | 1.14 (0.83 - 1.58) | 1.27 (1.03 - 1.58) \* |
| Siblings with the youngest child (yes, compared by no) | 1.95 (1.37 - 2.78)\* | 2.95 (2.29 - 3.78)\* |
| NICU4 admission of the youngest child (yes, compared by no) | 1.07 (0.63 - 1.81) | 0.65 (0.40 - 1.05) |
1: aPR stands for adjusted prevalence ratio. 2: 95% CI stands for 95% confidence interval. 3: NICU stands for neonatal intensive care unit. 4: K6 score stands for Kessler 6 scale score. * p<0.05 in Poisson regression analysis.
