# Supplementary material for: Association between parental recognition and engagement in child maltreatment: an Internet-based cross-sectional study in Japan
Source: Environ Health Prev Med. 2026 Mar 4;31:15. doi: 10.1265/ehpm.24-00388 (PMC12981977; doi:10.1265/ehpm.24-00388)
Supplement: Supplementary file 4 — Additional file 4: Table S4. Association between parental maltreatment behaviors and recognition status for subtypes by parental sex: psychological maltreatment (expanded results from Table 3). [file ehpm-31-015-s004.pptx]

## Slide 1
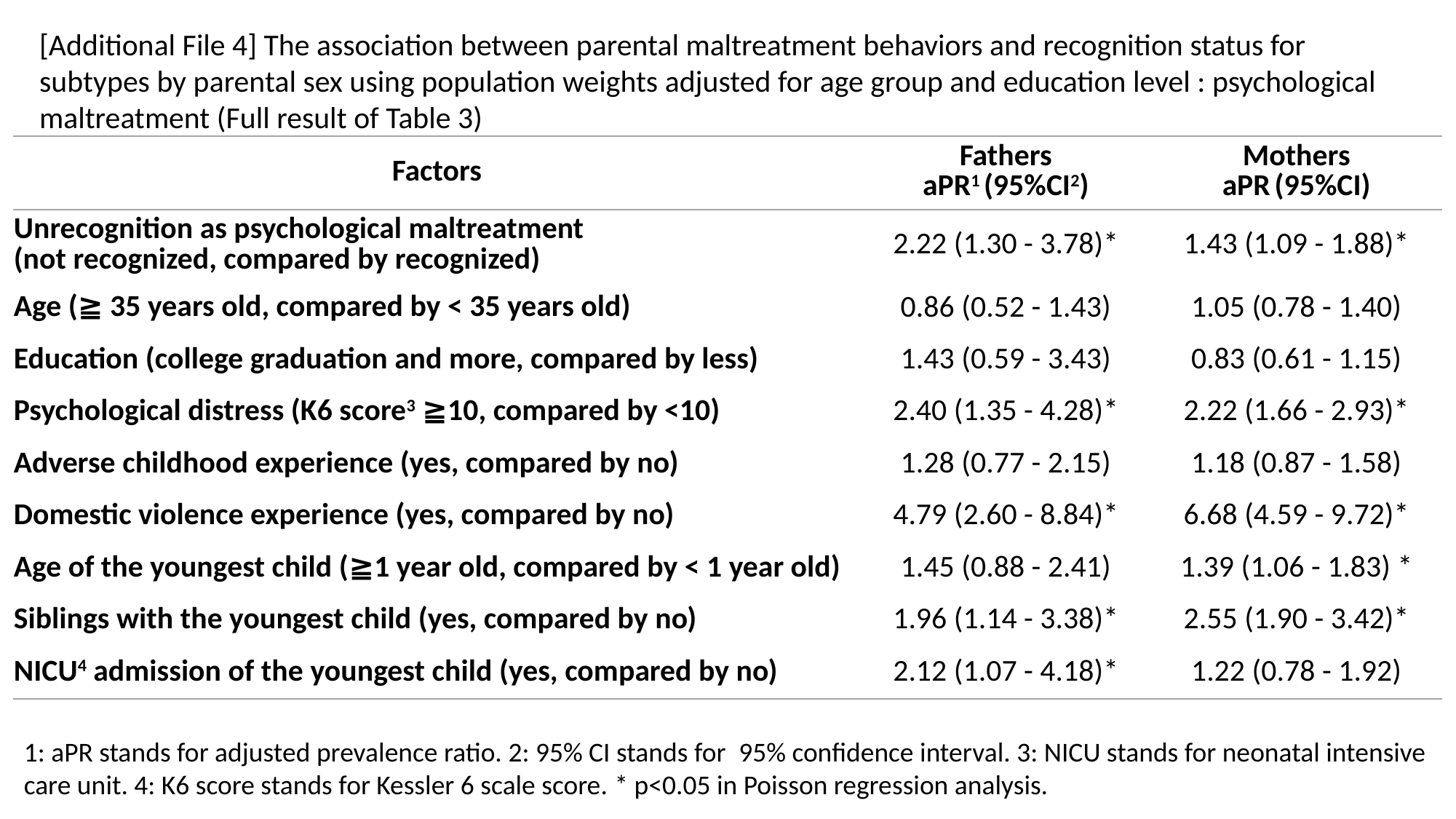

[Additional File 4] The association between parental maltreatment behaviors and recognition status for subtypes by parental sex using population weights adjusted for age group and education level : psychological maltreatment (Full result of Table 3)
| Factors | Fathers aPR1 (95%CI2) | Mothers aPR (95%CI) |
| --- | --- | --- |
| Unrecognition as psychological maltreatment (not recognized, compared by recognized) | 2.22 (1.30 - 3.78)\* | 1.43 (1.09 - 1.88)\* |
| Age (≧ 35 years old, compared by < 35 years old) | 0.86 (0.52 - 1.43) | 1.05 (0.78 - 1.40) |
| Education (college graduation and more, compared by less) | 1.43 (0.59 - 3.43) | 0.83 (0.61 - 1.15) |
| Psychological distress (K6 score3 ≧10, compared by <10) | 2.40 (1.35 - 4.28)\* | 2.22 (1.66 - 2.93)\* |
| Adverse childhood experience (yes, compared by no) | 1.28 (0.77 - 2.15) | 1.18 (0.87 - 1.58) |
| Domestic violence experience (yes, compared by no) | 4.79 (2.60 - 8.84)\* | 6.68 (4.59 - 9.72)\* |
| Age of the youngest child (≧1 year old, compared by < 1 year old) | 1.45 (0.88 - 2.41) | 1.39 (1.06 - 1.83) \* |
| Siblings with the youngest child (yes, compared by no) | 1.96 (1.14 - 3.38)\* | 2.55 (1.90 - 3.42)\* |
| NICU4 admission of the youngest child (yes, compared by no) | 2.12 (1.07 - 4.18)\* | 1.22 (0.78 - 1.92) |
1: aPR stands for adjusted prevalence ratio. 2: 95% CI stands for 95% confidence interval. 3: NICU stands for neonatal intensive care unit. 4: K6 score stands for Kessler 6 scale score. * p<0.05 in Poisson regression analysis.
