# Supplementary material for: Association between parental recognition and engagement in child maltreatment: an Internet-based cross-sectional study in Japan
Source: Environ Health Prev Med. 2026 Mar 4;31:15. doi: 10.1265/ehpm.24-00388 (PMC12981977; doi:10.1265/ehpm.24-00388)
Supplement: Supplementary file 5 — Additional file 5: Table S5. Association between parental maltreatment behaviors and recognition status for subtypes by parental sex: Neglect (expanded results from Table 3). [file ehpm-31-015-s005.pptx]

## Slide 1
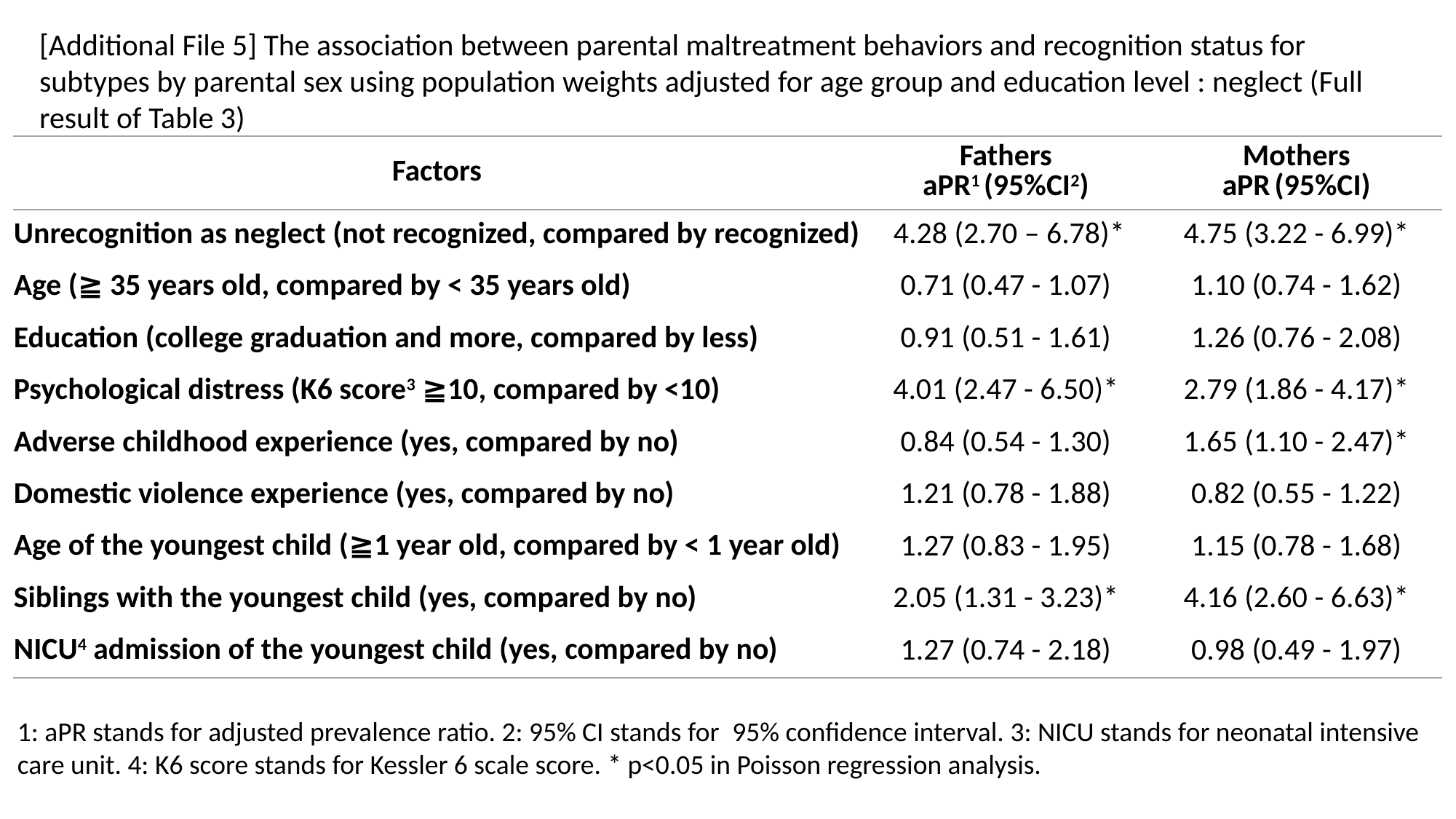

[Additional File 5] The association between parental maltreatment behaviors and recognition status for subtypes by parental sex using population weights adjusted for age group and education level : neglect (Full result of Table 3)
| Factors | Fathers aPR1 (95%CI2) | Mothers aPR (95%CI) |
| --- | --- | --- |
| Unrecognition as neglect (not recognized, compared by recognized) | 4.28 (2.70 – 6.78)\* | 4.75 (3.22 - 6.99)\* |
| Age (≧ 35 years old, compared by < 35 years old) | 0.71 (0.47 - 1.07) | 1.10 (0.74 - 1.62) |
| Education (college graduation and more, compared by less) | 0.91 (0.51 - 1.61) | 1.26 (0.76 - 2.08) |
| Psychological distress (K6 score3 ≧10, compared by <10) | 4.01 (2.47 - 6.50)\* | 2.79 (1.86 - 4.17)\* |
| Adverse childhood experience (yes, compared by no) | 0.84 (0.54 - 1.30) | 1.65 (1.10 - 2.47)\* |
| Domestic violence experience (yes, compared by no) | 1.21 (0.78 - 1.88) | 0.82 (0.55 - 1.22) |
| Age of the youngest child (≧1 year old, compared by < 1 year old) | 1.27 (0.83 - 1.95) | 1.15 (0.78 - 1.68) |
| Siblings with the youngest child (yes, compared by no) | 2.05 (1.31 - 3.23)\* | 4.16 (2.60 - 6.63)\* |
| NICU4 admission of the youngest child (yes, compared by no) | 1.27 (0.74 - 2.18) | 0.98 (0.49 - 1.97) |
1: aPR stands for adjusted prevalence ratio. 2: 95% CI stands for 95% confidence interval. 3: NICU stands for neonatal intensive care unit. 4: K6 score stands for Kessler 6 scale score. * p<0.05 in Poisson regression analysis.
